# Heterogeneity of influenza infection at precise scale in Yinchuan, Northwest China, 2012–2022: evidence from Joinpoint regression and spatiotemporal analysis

**DOI:** 10.1038/s41598-024-53767-w

**Published:** 2024-02-06

**Authors:** Lu Zhang, Yan Li, Ning Ma, Yi Zhao, Yu Zhao

**Affiliations:** 1 School of Public Health, Ningxia Medical University, Yinchuan, 750004 Ningxia China; 2 Ningxia Key Laboratory of Environmental Factors and Chronic Disease Control, Yinchuan, 750004 Ningxia China; 3 Yinchuan Center for Diseases Prevention and Control, Yinchuan, 750004 Ningxia China

**Keywords:** Diseases, Health care

## Abstract

Identifying high-risk regions and turning points of influenza with a precise spatiotemporal scale may provide effective prevention strategies. In this study, epidemiological characteristics and spatiotemporal clustering analysis at the township level were performed. A descriptive study and a Joinpoint regression analysis were used to explore the epidemiological characteristics and the time trend of influenza. Spatiotemporal autocorrelation and clustering analyses were carried out to explore the spatiotemporal distribution characteristics and aggregation. Furthermore, the hotspot regions were analyzed by spatiotemporal scan analysis. A total of 4025 influenza cases were reported in Yinchuan showing an overall increasing trend. The tendency of influenza in Yinchuan consisted of three stages: increased from 2012 to the first peak in 2019 (32.62/100,000) with a slight decrease in 2016; during 2019 and 2020, the trend was downwards; then it increased sharply again and reached another peak in 2022. The Joinpoint regression analysis found that there were three turning points from January 2012 to December 2022, namely January 2020, April 2020, and February 2022. The children under ten displayed an upward trend and were statistically significant. The trend surface analysis indicated that there was a shifting trend from northern to central and southern. A significant positive spatial auto-correlation was observed at the township level and four high-incidence clusters of influenza were detected. These results suggested that children under 10 years old deserve more attention and the spatiotemporal distribution of high-risk regions of influenza in Yinchuan varies every year at the township level. Thus, more monitoring and resource allocation should be prone to the four high-incidence clusters, which may benefit the public health authorities to carry out the vaccination and health promotion timely.

## Introduction

Influenza is an acute respiratory disease caused by the influenza virus, with the main symptoms of high fever, chills, and muscle or joint pain, which is the first infectious disease with global surveillance^1^. It is highly infectious and has quick transmission^2^. Influenza can aggravate the risk of corresponding complications, such as immune repression and intercurrent infection, and will bring a heavier disease burden to people^3,4^. According to the World Health Organization statistics, 3–5 million severe influenza cases are reported worldwide per year, and about 250–500 thousand cause death^5^. In 2021, more than 668 200 influenza cases were reported in China, accounting for 10.72% of the total infectious disease cases and the incidence is keeping increased^6,7^. Thus, influenza is still one of the serious public health problems in China threatening human health and has not yet been fully controlled, and deserves further studies.

Due to frequent variations in the influenza virus, new viral subtypes continue to emerge. Since 1947, the World Health Organization (WHO) has monitored influenza virus strains and gradually developed a global surveillance system^8^. Each influenza season, the WHO collected influenza virus samples from different regions for analysis and monitoring. Based on this data, the WHO identified the recommended influenza vaccine strains to address potential subtypes that may become prevalent in future influenza seasons. With the widespread implementation of influenza vaccines, many countries^9–12^ have seen a certain reduction in influenza incidence. However, in the past decade, many regions worldwide have reported influenza outbreaks, such as Thailand^13^, Italy^14^, Taiwan^15^, and America^16^. Previous studies^17–20^ on influenza largely focused on epidemiological characteristics and the analysis of influencing factors. In addition, Morabia^21^ found that variation in social and healthcare-based determinants exacerbates influenza epidemics, and low socioeconomic status (SES) individuals are prone to bear the burden of infection. Zhang et al.^22^ also suggested that the insufficient influenza vaccine supplement, antigenic shift, and expansion of influenza surveillance efforts might be the major causes of the dramatic changes in the outbreak. Diamond et al.^23^ found that within-year influenza patterns vary across mainland China concerning latitude and geographic location. However, there is limited research on associating epidemic characteristics with spatial clustering or spatiotemporal distribution features.

Some researchers^22,24,25^ have applied spatial techniques and geographic information systems to probe the geographic distribution characteristics and identify influenza clusters for high and low-risk regions and periods in China. Most of them emphasized the geographic heterogeneity of influenza at the urban level in economically developed cities. However, due to spatial heterogeneity, variations in economic development, and disparities in health resource allocation, the epidemiological characteristics of influenza differ across China^26^. Also, conducting research at the scale such as the city level may result in a lower spatial resolution compared to the finer resolution achievable at the township level. This could lead to a loss of detail in capturing the specific distribution and variations of diseases within smaller areas^27^. More precisely, at the township level, there may be specific local influencing factors such as demography, socio-cultural factors, health resources, etc., which might be overshadowed at the city or county level. Studying in a smaller geographical unit makes it easier to discover these local influencing factors. As one of the economically underdeveloped regions in Western China, Yinchuan City is the capital of the Ningxia Hui Autonomous Region, which has more densely populated communities and greater mobility of people than other cities in Ningxia. Moreover, Yinchuan has a temperate continental climate, with dry less rain, large temperature difference between day and night. Thus, this research aims to deepen the understanding of the geographic differences in influenza incidence in Western China, providing insights for targeted influenza prevention strategies and offering recommendations for the allocation such as vaccination and health resource allocation for policy-making departments.

## Materials and methods

### Study site

Yinchuan is situated at 37°29′ N–38°53′ N and 105°49′ E–106°53′ E and administers 61 townships across three districts (Xingqing, Jinfeng, Xixia), two counties (Yongning, Helan), and a county-level city (Lingwu), with a total area of 9025.38 km^2^. By the end of 2022, Yinchuan has a permanent resident population of 2,859,074. The specific information is as follows:

1. Xingqing district: includes Fenghuang North street, Jiefang West street, and other 15 townships.
2. Jinfeng district: includes Mancheng North street, Huanghe East street, and other eight townships.
3. Xixia district: includes Beijing West street, Wenchang street, and other nine townships.
4. Yongning County: includes Tuanjie West street, Yanghe township, and other nine townships.
5. Helan County: includes Xigang township, Jingui township, and other ten townships.
6. Lingwu: includes Haojiaqiao township, Chongxing township, and other ten townships.

### Data collection

The case data from 2012 to 2022 in Yinchuan City were obtained from the National Monitoring and Reporting Management System for Communicable Disease. All cases were diagnosed based on the clinical symptoms and laboratory test results according to the diagnostic guidelines issued by the Chinese Ministry of Health^1^. Demographic data were obtained from the Ningxia Statistical Yearbook and statistical bulletins of counties and districts (http://nxdata.com.cn/publish.htm?cn=G01). The town-level vector map of Yinchuan was derived from the Chinese National Fundamental Geographic Information System (http://www.ngcc.cn/ngcc/). All cases were desensitized, geocoded by the specific administrative code, and matched to the town-level layers.

## Methods

### Descriptive analysis

We described the epidemic characteristics of influenza in Yinchuan from 2012 to 2022, including reported incidence, reported number, onset time, gender, age, occupation, and regional distribution. We use the annual population composition and influenza incidence rate in each township of Yinchuan to calculate the standardized morbidity ratio (SMR) of influenza. The formula shows as follows:

1 $$SMR=\frac{{y}_{it}}{{E}_{it}},$$

where ${y}_{it}$ is the number of reported cases in township $i(1\le i\le 61)$ during year $t(2012\le t\le 2022)$ and ${E}_{it}$ is the expected cases in township $i$ during year $t$, which can be calculated by multiplying the annual population composition and the reported incidence of influenza in the county for a particular year $t$.

### Joinpoint regression analysis and cross-correlation function (CCF) analysis

Joinpoint regression analysis was created by the National Cancer Institute (NCI)^28,29^ to analyze trend changes, primarily applied in the analysis of time series data. In our study, we used the sequence of observations (${t}_{1},{y}_{1}$),…… (${t}_{n},{y}_{n}$) , (${t}_{i}\le \dots \le {t}_{n}$) ,where $t$ is the year from 2012 to 2022), ${\text{y}}$ is the cases of influenza and $n$ is the period of the study to establish the regression model:

2 $$E\left[\left.y\right|t\right]={e}^{{\beta }_{0}+{\beta }_{1}t+{\delta }_{1}{\left(t-{\tau }_{1}\right)}^{+}+\dots +{\delta }_{k}{\left(t-{\tau }_{k}\right)}^{+}},$$

where $k$ is the number of turning points, ${\tau }_{k}$ is the unknown turning point, ${\beta }_{0}$ is the invariant parameter, ${\beta }_{1}$ is the regression coefficient, ${\delta }_{k}$ is the regression coefficient of the segment $k$. When $\left(t-{\tau }_{k}\right)>0$, ${\left(t-{\tau }_{k}\right)}^{+}=t-{\tau }_{k}$, otherwise ${\left(t-{\tau }_{k}\right)}^{+}=0$.

Due to APC being calculated based on annual data, it provides a high-level overview of trends at the annual scale and may smooth out fluctuations due to seasonality or periodicity, resulting in an annual trend that appears more consistent upward. To analyze the data at different time scales, we also fitted the model by the monthly and annual cases, different ages, and different regions in Yinchuan successively. Using the annual percent change (APC), monthly percent change (MPC), average annual percent change (AAPC), and their 95% confidence intervals (CI) to describe each line segment change, the change between the inflection point and the change over the entire period, respectively. The calculation formulas are shown as follows:

3 $$APC=\left[\frac{{y}_{n+1}-{y}_{n}}{{y}_{n}}\right]=\left\{{e}^{{\beta }_{1}}-1\right\}\times 100,$$

4 $$AAPC=(\frac{{\sum }_{i=1}^{k}\left({APC}_{i}\times {\omega }_{i}\right)}{{\sum }_{i=1}^{k}{\omega }_{i}}),$$

where ${APC}_{i}$ is the $APC$ for the ${i}$ th segment, ${\omega }_{i}$ is the length of each segment in the range of years. MPC denotes the monthly percentage change, with a calculation formula identical to that of APC, whose data pertains to monthly records. The method of cross-correlation function (CCF) analysis was given in Supplementary Material.

### Trend surface analysis

By fitting a two-dimensional non-linear regression function using the least squares method, we performed a Three-Dimensional Trend Surface Analysis to help visualize influenza spatial variations across different geographical locations^30^. The analysis decomposes the observed values, such as the incidence of influenza, into three components: local anomalies, regional trends and random errors. Based on the SMR of influenza from 61 townships in Yinchuan from 2012 to 2022, we calculate the average SMR value for each township as the dependent variable (Z-axis), with the geographical coordinates (latitude and longitude) of each township as the independent variables (X-axis and Y-axis). Then we conduct a spatial three-dimensional trend surface analysis to explore the overall distribution of influenza in Yinchuan.

Within the graphical representation, disease indicator data points are meticulously projected onto two-dimensional planes (XZ plane and YZ plane). Through data fitting, the estimates derived reflect the nuanced changing trends of the studied phenomenon across the entire region. Employing polynomial fitting for three-dimensional data projection onto discrete points, on the XZ plane aligned with the Y-axis, the line illustrates the disease index trend variations in the north–south direction (latitude), with the arrow indicating northward. Similarly, on the YZ plane aligned with the X-axis, the line depicts the trend changes in the east–west direction (longitude), with the arrow indicating eastward^31^.

### Spatial auto-correlation analysis

The global and local spatial autocorrelation method is used to explore the overall aggregation of the study area and locate the specific area. Moran’s* I* was used to investigate the autocorrelation of global space, the value range is from −1 to 1. Statistically significant positive or negative values indicate positive or negative spatial autocorrelation and zero values indicate random spatial autocorrelation^32^. The Moran’s index $I$ is calculated as follows:

5 $$I=\frac{m{\sum }_{i=1}^{m}{\sum }_{j=1}^{m}{\omega }_{ij}\left({x}_{i}-\overline{x}\right)\left({x}_{j}-\overline{x}\right)}{\left({\sum }_{i=1}^{m}{\sum }_{j=1}^{m}{\omega }_{ij}\right){\sum }_{i=1}^{m}{\left({x}_{i}-\overline{x}\right)}^{2}},$$

where $m$ is the number of geographic regions, ${x}_{i}$ is the number of reported cases in $i$-th spatial feature, ${x}_{j}$ is the number of reported cases in $j$-th spatial feature, $\overline{x}$ is the mean of the variable $x$ across all geographic regions, ${\omega }_{ij}$ is a spatial weight matrix (when $i$ is adjacent to $j$, ${\omega }_{ij}=1$ otherwise ${\omega }_{ij}=0$).

Local Indicators of Spatial Autocorrelation (LISA) analysis is commonly used to explore specific aggregation patterns and regions^33,34^. The local Moran's *I* statistic is usually used to evaluate whether a feature at a particular location is correlated with its neighboring features and helps identify spatial clusters of high or low values and classifies locations into four categories: High–High (H–H), High–Low (H–L), Low–High (L–H), and Low–Low (L–L). These categories provide insights into whether similar or dissimilar values are clustered together in the spatial domain. The local Moran’s index* I* is calculated as follows:

6 $${I}_{i}=\frac{{x}_{i}-\overline{x}}{{\sum }_{j=1}^{m}{\omega }_{ij}\left({x}_{j}-\overline{x }\right)},$$

where ${I}_{i}$ is the local Moran’s index at geographical unit $i$, the interpretations of the other parameters in Eq. (5) as above.

### Spatiotemporal scan analysis

Kulldorff’s scan statistics were applied to detect clusters of influenza cases in space–time settings^35^. Its sensitivity drives surveillance systems to detect outbreaks earlier than before. The method has strong statistical robustness and interpretability of the analytical results^36^. In practice, we create a scan window to explore the time of disease clusters by the log-likelihood ratio (LLR). If there is a larger LLR value and a smaller *P* value, we consider that the region is likely to be a high cluster area, and the risk can be evaluated by relative risk (RR). Scan analysis can not only reveal the law of the aggregation area in the geographical space with time, but also obtain the relative risk, and locate the position of the spatial aggregation area more accurately^37–39^.

### Statistical software

Descriptive analysis was generated by SPSS 26.0, Joinpoint regression analysis was carried out by Joinpoint Program 5.0.2, trend surface analysis was completed by ArcGIS10.6, spatial auto-correlation analysis was performed by GeoDa 1.10 (Arizona State University, Phoenix, AZ, USA) and ArcGIS 10.6, whose number of Monte Carlo random repeated simulations was set to M = 999. Spatiotemporal scan analysis was performed by SaTScan 9.4.1 (Martin Kulldorff, Boston, MA, USA). *P* < 0.05 was considered statistically significant.

## Results

### Epidemiological characteristics

A total of 4025 influenza cases were reported in Yinchuan from 2012 to 2022, with an average annual incidence of 15.47/100,000, which is lower than that of the national average level. Fig.S1. shows the spatial and temporal distribution characteristics of influenza in Yinchuan from 2012 to 2022, and the spatial and temporal analysis at the township level may be beneficial to identify more high-risk areas. Except Lingwu was decreasing, the incidence in Xingqing, Jinfeng, Xixia, Yongning, and Helan was gradually increasing, among which Jinfeng with the fastest increase rate. Spatially, 1603 cases were in Jinfeng with an average annual incidence of 35.82 per 100,000, followed by Xingqing (1603, 13.37/100,000), Xixia (623, 14.93/100,000), Yongning (309, 10.80/100,000), Helen (148, 487/100,000) and Lingwu (233, 7.36/100,000).

**Figure 1:**
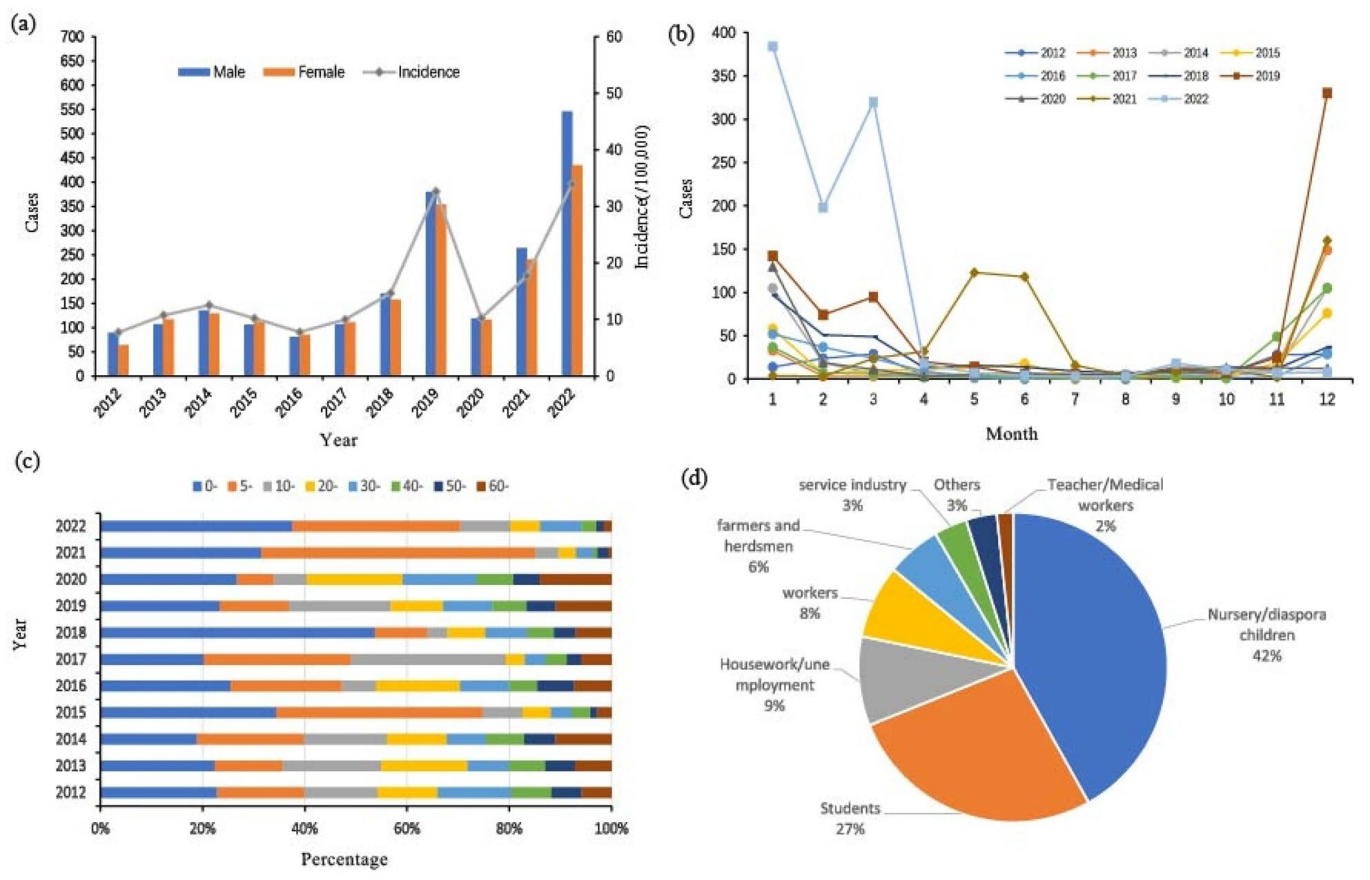
Epidemiological features of influenza in Yinchuan, China, 2012–2022: (**a**) distribution of annual influenza incidence by gender; (**b**) monthly distribution of influenza; (**c**) influenza distribution by age; (**d**) influenza distribution by occupation.

From 2012 to 2022, the tendency of influenza in Yinchuan consisted of three stages: increased from 2012 to the first peak in 2019 (32.62/100,000) with a slight decrease in 2016; during 2019 and 2020, the trend was downwards; then it increased sharply again and reached another peak in 2022, with the incidence of 33.96/100,000 (Fig. 1a). The seasonal trend analysis displayed there was a unimodal mode in Yinchuan, the peak appeared from November to March (as shown in Fig. 1b).

Among these Influenza cases, 2103 (52.2%) were male and were distributed in all age groups, mainly in 0-(30.7%) and 5-(25.9%). It was worth noting that the cases under 19 account for 68.9% (Fig. 1c). In terms of occupational distribution, as shown in Fig. 1d, nursery, and diaspora children were the main group (41.9%), followed by students (27%) and housework and unemployed people (9.3%).

### Joinpoint regression analysis and cross-correlation function (CCF) analysis

**Figure 2:**
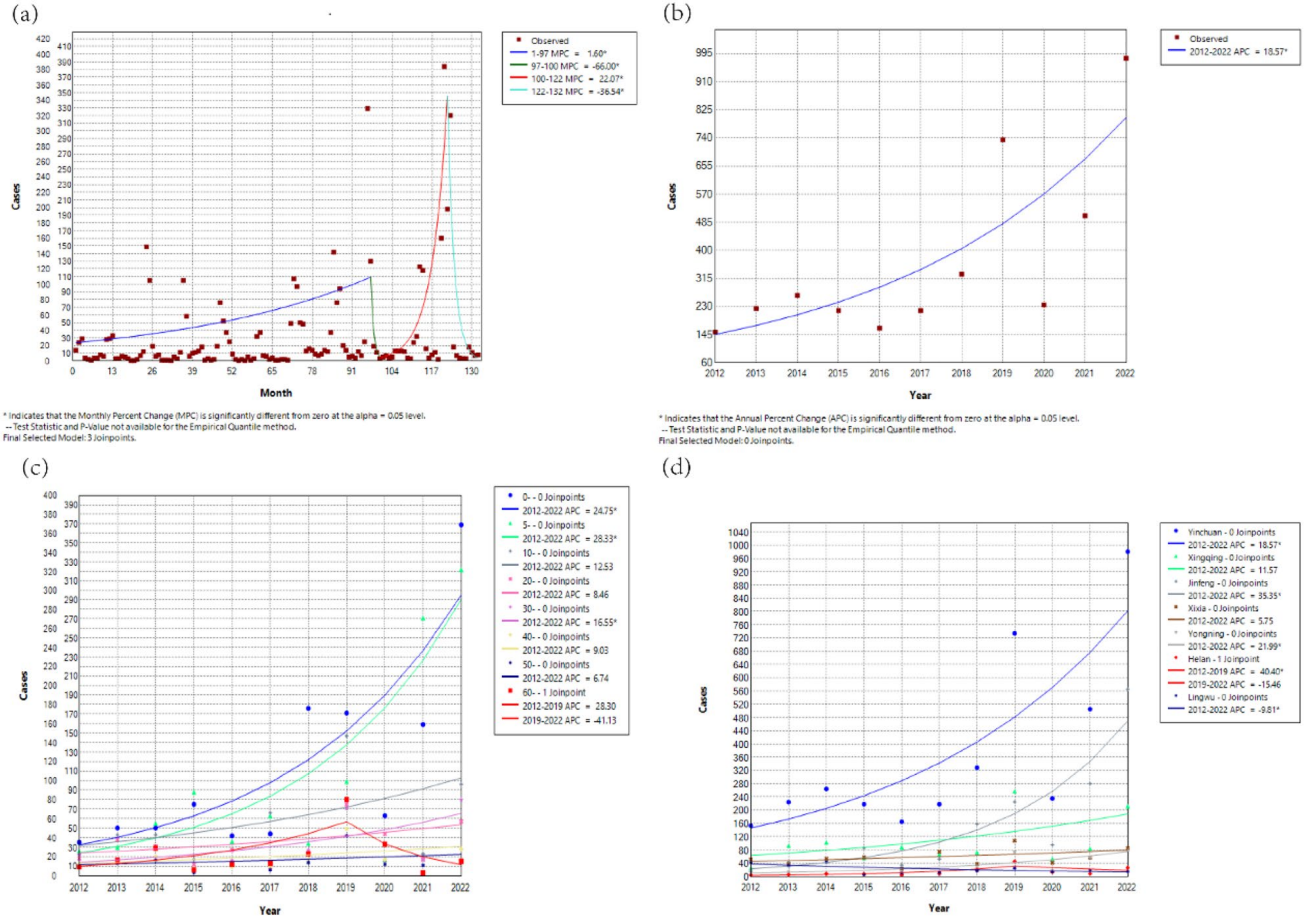
Changing trend of influenza in Yinchuan, China, 2012–2022: (**a**) changing trend of monthly cases of influenza in Yinchuan, 2012–2022; (**b**) changing trend of annual cases of influenza in Yinchuan, 2012–2022; (**c**) change in influenza cases in different age groups in Yinchuan, 2012–2022; (**d**) change in influenza cases at county level in Yinchuan, 2012–2022.

Figure 2. shows the changing trend of influenza in Yinchuan. The results found that there were 3 turning points from Jan. 2012 to Dec. 2022, namely the 97th month (January 2020), the 100th month (April 2020), and the 122nd month (February 2022), respectively. A "rise-fall-rise" trend was observed, and the MPC of four segments was statistically significant, as shown in Fig. 2a and Table 1. The annual cases of influenza from 2012 to 2022 appeared an upward trend (APC = 18.57, P < 0.05) (Fig. 2b). The children under 10 years old displayed an upward trend and were statistically significant. Among all groups, the 5–10 group is rising the fastest. One turning point was presented in the 60-year-old group, which displayed a “first rise then descend” trend (before 2019 APC was 28.30 and after 2019 APC was -41.13, respectively), other groups were the same as the overall trend (Fig. 2c). The cases showed an overall upward trend and were statistically significant in Yinchuan. Helan County had a turning point in 2019 (Table S1), while other regions showed a single upward trend, among them Lingwu presented a distinctive downward trend (Fig. 2d). In the cross-correlation function (CCF) analysis between influenza cases and influenza vaccination and Baidu index time series data, a strong positive correlation was observed, as shown in Fig. S2.

**Table 1:** Joinpoint analysis results of monthly reported cases of influenza in Yinchuan, 2012–2022.

Segment	Turning point	Time (year/month)	MPC (%)	95% CI (%)
1		2012/1–2020/1	1.60*	1.32 to 2.61
2	2020/1	2020/1–2020/4	−66.00*	−80.84 to 35.59
3	2020/4	2020/4–2022/2	22.07*	20.97 to 41.80
4	2022/2	2022/2–2022/12	−36.54*	−55.50 to 28.70

*The monthly percent change (MPC) is significantly different from 0 at the $\mathrm{\alpha }$ = 0.05.

### Spatial distribution and trend surface analysis

**Figure 3:**
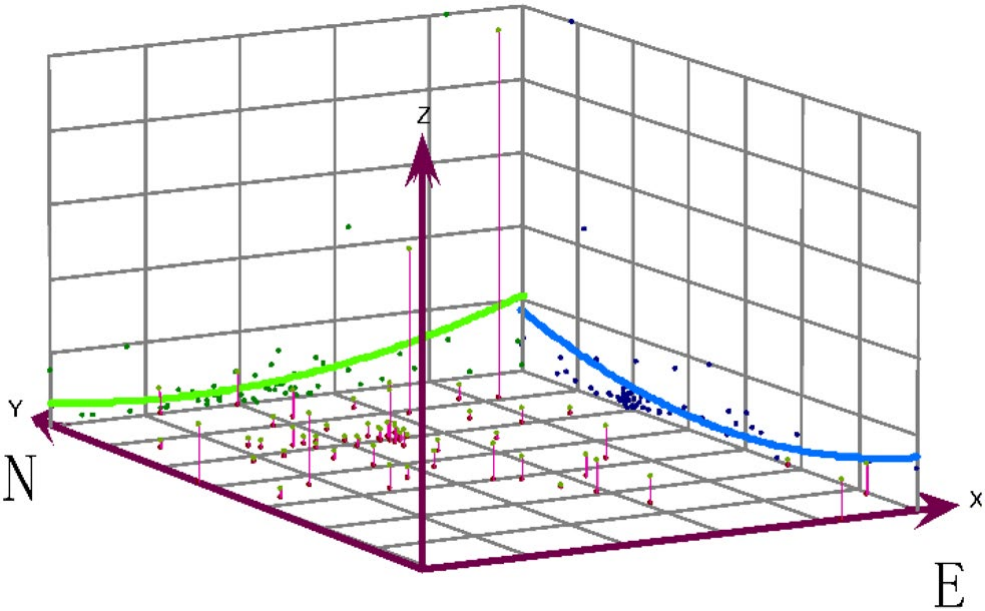
Spatial three-dimensional trend surface analysis of influenza.

In Fig. S3, we observed that the annual average incidence of influenza presented a significant spatial heterogeneity at the township level, and there was a shifting trend from northern to central and southern Yinchuan. According to the three-dimensional trend surface analysis (Fig. 3), the results also validated that the standardized morbidity ratio (SMR) of influenza in the North is slightly higher than that in the South, the Eastern is higher than that in the Western, and the central region was lowest.

### Spatiotemporal clustering analysis

**Figure 4:**
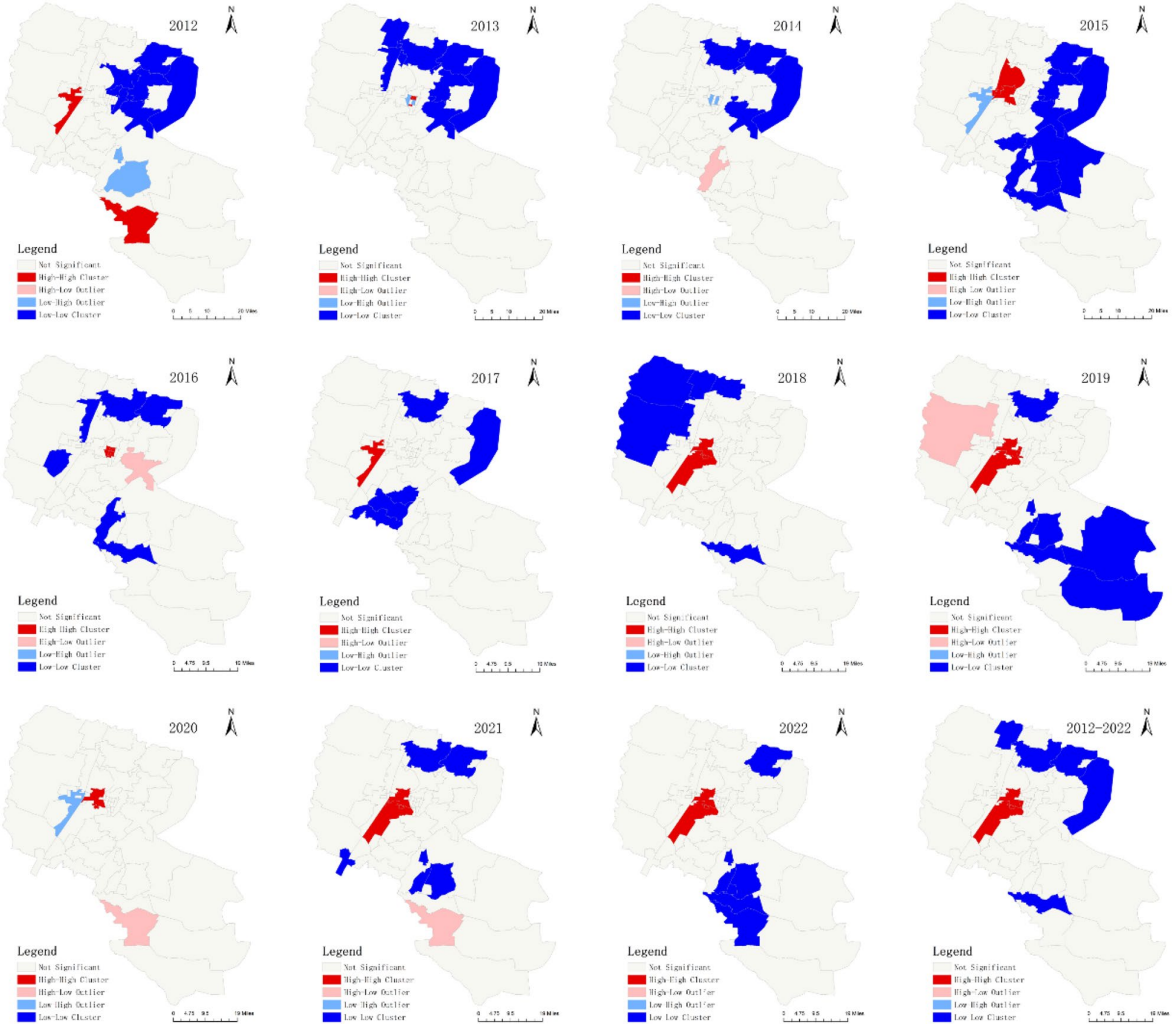
Yearly local spatial auto-correlation of influenza at the township level in Yinchuan, China, 2012–2022.

Global auto-correlation analysis indicated that the distribution of influenza presented a significant positive spatial auto-correlation at the township level since 2015, as displayed in Table S2. In Table S3 and Fig. 4, LISA analysis of influenza from 2012 to 2022 was performed to detect the hot spots in Yinchuan, and four hot spots of influenza were identified: Funing Street, Changcheng Middle Road, ShanghaiWest Road, Liangtian Town. The high-high cluster was mainly located in the central of Yinchuan, such as Changcheng Middle Road, Shanghai West Road, Liangtian Town, etc. The cases in cold spots were significantly lower than those in hot spots (${\chi }^{2}$=656.96, ${\rm P}$<0.0001). The specific results are shown in Table S4. Another interesting fact was observed that the high-high cluster in 2016 covered the greatest number of towns, but the incidence was relatively lower.

### Spatiotemporal aggregation scan analysis

**Figure 5:**
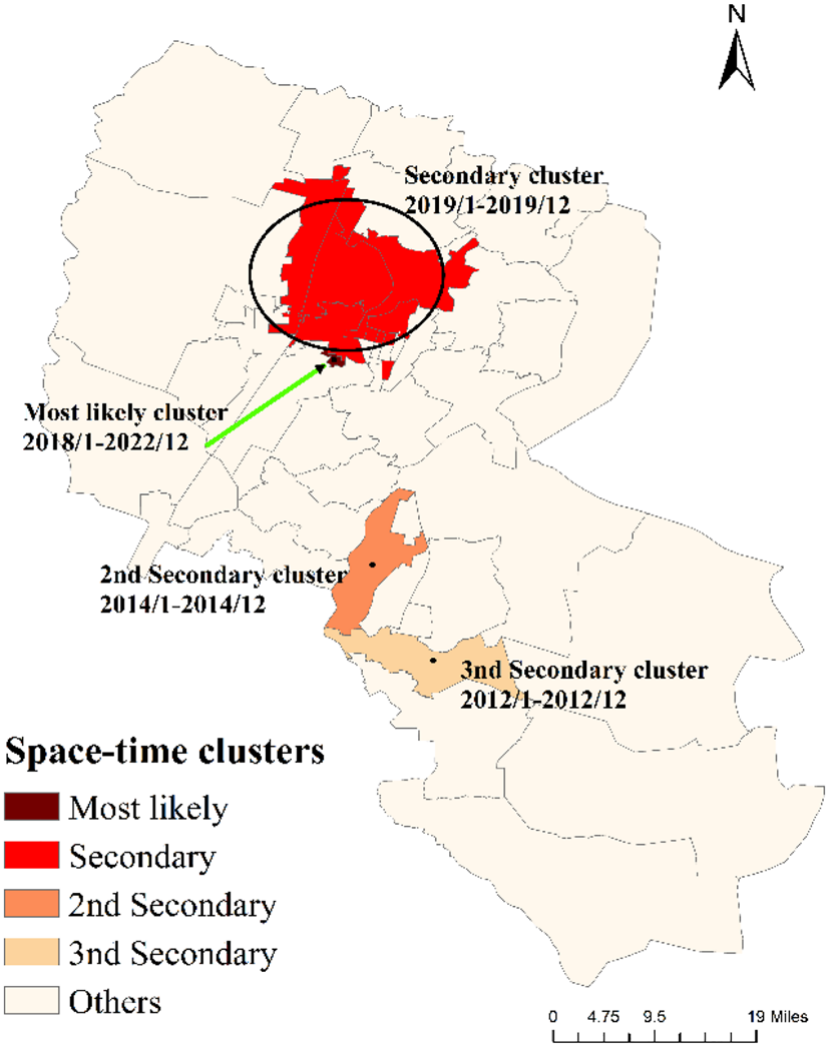
Spatiotemporal clusters of influenza incidences at the township level in Yinchuan, China, 2012–2022.

The results of spatiotemporal aggregation scan statistics are in Fig. 5 and Table S5. We found that there were four high-incidence clusters of influenza. The most likely cluster was Changcheng Middle Road from January 2018 to December 2022 (RR = 7.72, LLR = 1031.70, *P* < 0.0001). Followed by Fenghuang North Street with a radius of 11.37 km (RR = 2.42, LLR = 75.99, *P* < 0.0001). In addition, Wutongshu Town (RR = 14.47, LLR = 64.28, *P* < 0.0001) and Chongxing Town (RR = 5.30, LLR = 26.46, *P* < 0.0001) were also the two high-incidence clusters of influenza in Yinchuan.

## Discussion

World Health Organization reported that 290,000–650,000 people dead from influenza-related respiratory diseases worldwide per year, accounting for 8.2% of all respiratory disease deaths, causing a heavy social and economic burden^40^. As influenza is an airborne disease, vaccination is the most effective way to prevent infection and severe outcomes caused by influenza viruses. However, the influenza vaccine (such as LAIV3, IIV4, etc^1^) is a non-immunization program vaccine in most areas of China, and residents are vaccinated voluntarily, thus, accurately identifying the high-risk clusters and susceptible populations of influenza may provide a scientific basis for the planning for the influenza vaccines supplies as well as provision of other respective health care resources.

The analysis found that the overall trend of influenza in Yinchuan was increasing from 2012 to 2019, which was consistent with the tendency of influenza in China^41^. Influenza reached its first peak period in 2019, with an incidence of 32.62/100,000. Then, we observed a remarkable decline in 2020, staying in step with the United States, Singapore, and China^42–44^. After nearly two years, influenza up-resurgence and reached another peak in 2022, as was reported in several studies before^15^. In 2019, the National Health Commission of the People's Republic of China released the "Influenza Diagnosis and Treatment of Influenza (2019)"^45^, which standardized the diagnosis, treatment, and reporting procedures of influenza, resulting in an increase in the reporting rate. In 2020, with the outbreak of the 2019 novel coronavirus disease (COVID-19), Ningxia initiated a Level 1 response (Feb.2020) to a major public health emergency under relevant national laws and regulations. The results of CCF analysis also indicated that influenza cases have a strong positive correlation between vaccination and public health awareness. Some researchers^12,46,47^ have come to the same conclusion. Non-pharmaceutical interventions (NPIs), such as mask-wearing, social distancing, travel restrictions, patient isolation, and other public health measures have limited people's exposure to the influenza virus, leading to a downward trend^42,48^. Since 2021, Ningxia achieved significant success against the epidemic. The response level was gradually adjusted to Level 2 and Level 3 (March and May 2020). Due to the prolonged absence of exposure to the pathogenic microorganism, there is an increasing number of individuals lacking immunity to the influenza virus^49,50^. Wang et al.^51^ quantified the population susceptibility to influenza viruses has increased by 45.1–72.9% after the COVID-19 pandemic. Thus, once pandemic prevention measures are lifted, there is a significant surge. Such an "Immunity debt" phenomenon was also observed in other respiratory syncytial virus^49,52,53^.

Regarding seasonal trends, influenza in China varies with latitude and shows diverse spatial patterns and seasonal characteristics^23^. Similar to other reports^25,33^, influenza in Yinchuan displayed an unimodal mode, with the peak appearing from December to March. Notably, the low influenza incidence appeared in July and August, in keeping with the school holiday and population mobility peak. The subpopulation analysis showed that nursery and diaspora children, students, housework, and unemployed workers were at high risk of influenza as well. Males were more susceptible than females, which may be related to different behavior patterns. Males often have more social pressure, work intensity, and outdoor activities, which would increase exposure and facilitate virus transmission^54,55^. Previous studies^56–58^ have indicated that confined spaces like schools and families accelerated the spread of the influenza virus and increased the possibility of infection among members. Beyond that, children under 19 years old accounted for over half of all cases (68.9%). Existing research demonstrated that school-based influenza vaccination can effectively reduce the incidence of influenza among school-age children. Lau et al.^59^ suggested that the incidence of vaccinated students was significantly reduced compared with those not vaccinated (7.7% vs.14.1%). Thus, kindergartens and schools should strengthen health education, and timely provide school-based influenza vaccination to effectively reduce influenza infections.

Based on the Joinpoint regression analysis, the overall trend of influenza has been increasing from 2012 to 2022. Notably, we identified three turning points whose timings closely align with the implementation of NPIs in Ningxia. Previous studies^42,48^ observed similar trends in other respiratory diseases. Therefore, we have reasons to believe that NPIs significantly influence the incidence of influenza. Regarding different age groups, a single increasing trend over the entire period was displayed except for the age group of 60 years. Groups 5–9 years, 0–4 years, and 30–40 years groups exhibited a relatively higher APC among all groups, ranking the top three. During the peak influenza season, the annual infection rate of influenza in children under ten years old can reach 50%^60,61^. Individuals in the 30–40 years group often have more frequent interactions with others in career development, and the pressures of work can also impact the immune system, thereby increasing the chances of influenza transmission^54^. When stratified by region, Lingwu showed a decreasing trend, while other counties generally exhibited an upward trend. Among them, Jinfeng County experienced the fastest growth. Mamelund^62^ and Munday^63^ indicated that socioeconomic conditions influence influenza incidence. According to Yinchuan Statistical Yearbook^64^, Jinfeng District has better socioeconomic conditions compared to other counties. The high population density in this area facilitates the spread of the virus and the numerous medical institutions lead to a high diagnostic reporting rate. Those potentially contributing to its higher incidence compared to other counties.

Global auto-correlation analysis and spatiotemporal scan statistics found that the distribution of influenza presented a significant positive spatial auto-correlation at the township level and four high-incidence clusters of influenza were detected in Yinchuan. The most likely cluster was Changcheng Middle Road with 7.72 times the risk than that outside the cluster area, and the 14 districts in the second spatial–temporal cluster principally concentrated in Xingqing and Jinfeng. The streets in Yinchuan possess different socioeconomic levels, infrastructure, and healthcare conditions, which might affect the spatiotemporal variations. Several studies have used spatial analysis to explore the distribution of influenza in many regions of China, such as Shanghai city^65^, Shandong province^66^, Guangzhou province^24^, and mainland China^18^. However, most of them are focused on a larger scale to analyze epidemical tendency, such as provincial and district level, there has been no report on the township level to study spatial epidemiological of influenza. As townships are the smallest units implementing basic public health services, their results are more targeted and geographically precise. The spatiotemporal clustering analysis result showed that the cases in cold spots were significantly lower than those in hot spots. Prevention is more cost-effective than treatment. Although increased attention and resources may be required in hot spots, cold spots should not be ignored to prevent potential outbreaks as well. Consider the following measures to strengthen surveillance in non-hotspot areas may be helpful. First, provide training for healthcare institutions and professionals and encourage healthcare workers to report unusual cases more diligently. Second, encourage residents to proactively report symptoms and conduct community outreach and education activities to promote self-protection. Third, set up health emergency response mechanisms to ensure a quick and organized response once disease outbreaks. Finally, incorporate modern technologies such as artificial intelligence and big data analysis to enhance monitoring effectiveness.

There are some reasons that may be responsible for the heterogeneity of influenza infection at a precise scale in Yinchuan, we first carried out a spatial regression model to capture the spatial correlation between the vaccine coverage and influenza incidence, as shown in Table S6 in supplementary material, including the Spatial Lag Model (SLM), Spatial Error Model (SEM), and Spatial Dubin Model (SDM). However, due to the results of Lagrange Multiplier (LM) tests being non-significant (P > 0.05), we subsequently conducted an Ordinary Least Squares (OLS) regression analysis and found that vaccine coverage was significantly negatively associated with influenza incidence (β = −1.3558, P < 0.05), which implied that vaccination may be an effective protective measure to reduce influenza incidence, see Table S7 for more details.

Our results have important implications for the planning and optimization of immunization programs and healthcare provision at a finer scale. However, this study also has some limitations. First, we found that the number of vaccinations is associated with the incidence of influenza in Yinchuan City, however, due to the limited data source of economic factors, medical and health resources, etc. at the township scale, more influencing factors of the spatiotemporal variations of influenza at the township should be performed quantificationally. Second, the data of analysis sourced from the surveillance system, misdiagnosis, and misreporting are unavoidable because it is difficult to distinguish influenza from other respiratory viruses without laboratory testing^22^. Third, as mentioned above, health behavior and awareness of residents is one of the effective ways to prevent influenza, thus, carrying out fine-scale spatiotemporal surveillance results are more targeted and geographically precise. Fourth, prevention is more cost-effective than treatment. So more accurate disease monitoring and reporting enable local health authorities to swiftly identify and respond to potential outbreaks, which can mitigate these impacts and reduce their impact on the local economy. It also can guide the precise allocation and deployment of medical resources, enhancing the effectiveness of medical services and reducing unnecessary costs.

## Conclusions

The study described the epidemiological characteristics of influenza and detected hotspot regions at the township level in Yinchuan, Ningxia, from 2012 to 2022. The tendency of influenza in Yinchuan consisted of three stages: increased from 2012 to the first peak in 2019 (32.62/100,000) with a slight decrease in 2016; during 2019 and 2020, the trend was downwards; then it increased sharply again and reached another peak in 2022, with the incidence of 33.96/100,000. Influenza in Yinchuan displayed an unimodal mode within 1 year, with the peak appearing from December to March. The Joinpoint regression analysis found that there were three turning points from January 2012 to December 2022, namely January 2020, April 2020, and February 2022. The children under ten displayed an upward trend and were statistically significant. The trend surface analysis indicated that there was a shifting trend from northern to central and southern. Global auto-correlation analysis and spatiotemporal scan statistics found that the distribution of influenza presented a significant positive spatial auto-correlation at the township level and four high-incidence clusters of influenza were detected in Yinchuan. Different measures for different regions should be guaranteed for effective prevention strategies such as vaccination and health resource allocation.

### Supplementary Information

Supplementary Information.

## Data Availability

The datasets used and/or analyzed during the current study are available from the corresponding author upon reasonable request.
