# Supplementary material for: Heterogeneity of influenza infection at precise scale in Yinchuan, Northwest China, 2012–2022: evidence from Joinpoint regression and spatiotemporal analysis
Source: Sci Rep. 2024 Feb 6;14:3079. doi: 10.1038/s41598-024-53767-w (PMC10847441; doi:10.1038/s41598-024-53767-w)
Supplement: Supplementary file 1 — Supplementary Information. [file 41598_2024_53767_MOESM1_ESM.docx]

Appendix A. Supplementary material

**Table S1** Joinpoint regression analysis results of influenza in Yinchuan, 2012−2022

| Region | Whole Year | | |  | Section 1 | | |  | Section 2 | | | |
| --- | --- | --- | --- | --- | --- | --- | --- | --- | --- | --- | --- | --- |
|  | year | AAPC  (%) | 95%*CI*(%) |  | Start-stop year | APC  (%) | 95%*CI*(%) |  | Start-stop year | APC(%) | 95%*CI*(%) |  |
| Yinchuan | 2012-2022 | 18.57* | 7.50-37.75 |  | 2012-2022 | 18.57* | 7.50-37.75 |  |  |  |  |  |
| Xingqing | 2012-2022 | 11.57 | -2.51-33.77 |  | 2012-2022 | 11.57 | -2.51-33.77 |  |  |  |  |  |
| Jinfeng | 2012-2022 | 35.35* | 26.62-57.69 |  | 2012-2022 | 35.35* | 26.62-57.69 |  |  |  |  |  |
| Xixia | 2012-2022 | 5.75 | -3.92-18.36 |  | 2012-2022 | 5.75 | -3.92-18.36 |  |  |  |  |  |
| Yongning | 2012-2022 | 21.99* | 9.04-54.32 |  | 2012-2022 | 21.99* | 9.04-54.32 |  |  |  |  |  |
| Helan | 2012-2022 | 20.58* | 4.57-82.34 |  | 2012-2019 | 40.40* | 15.13-1005.88 |  | 2019-2022 | -15.46 | -66.84-29.38 |  |
| Lingwu | 2012-2022 | -9.81* | -19.18-2.24 |  | 2012-2022 | 9.81* | -19.18-2.24 |  |  |  |  |  |

* Indicates that the average annual percent change (AAPC) and annual percent change (APC) are significantly different from 0 at the α = 0.05.

**Table S2** Global spatial auto-correlation test of influenza in Yinchuan, China, 2012–2022

| Year | Moran’s *I* | $P$-value | *Z*-score |
| --- | --- | --- | --- |
| 2012 | 0.0743 | 0.093 | 1.3386 |
| 2013 | 0.0754 | 0.055 | 1.8118 |
| 2014 | 0.0083 | 0.186 | 0.4023 |
| **2015** | **0.3277** | **0.002** | **4.8323** |
| **2016** | **0.2536** | **0.001** | **6.1502** |
| **2017** | **0.1222** | **0.048** | **2.0417** |
| **2018** | **0.3065** | **0.001** | **4.5510** |
| **2019** | **0.2996** | **0.003** | **4.0538** |
| **2020** | **0.2586** | **0.001** | **4.1016** |
| **2021** | **0.1517** | **0.004** | **3.9953** |
| **2022** | **0.1879** | **0.004** | **4.6592** |

**Table S3** High–high areas (hot spots) detected in the LISA analysis for influenza from 2012 to 2022

| Year | Towns/streets | | Hot-spot lists |
| --- | --- | --- | --- |
| 2012 | 2 | Xingjing Town, Haojiaqiao Town | |
| 2013 | 3 | Culture Street, Yuhuangge North Street, Qianjin Street | |
| 2014 | 0 |  | |
| 2015 | 4 | Mancheng North Street, Shanghai West Road, Fengdeng Town, Xihuayuan Road | |
| 2016 | 7 | Jiefang West Street, Cultural Street, Funing Street, Xinhua Street, Yuhuangge North Street, Qianjin Street, Zhongshan South Street | |
| 2017 | 1 | Xingjing Town | |
| 2018 | 3 | Changcheng Middle Road, Shanghai West Road, Liangtian Town | |
| 2019 | 5 | Jiefang West Street, Funing Street, Qianjin Street, Shanghai West Road, Liangtian Town | |
| 2020 | 2 | Changcheng Middle Road, Shanghai West Road | |
| 2021 | 4 | Huanghe East Road, Changcheng Middle Road, Shanghai West Road, Liangtian Town | |
| 2022 | 3 | Changcheng Middle Road, Shanghai West Road, Liangtian Town | |
| Average | 4 | Funing Street, Changcheng Middle Road, Shanghai West Road, Liangtian Town | |

**Table S4** Comparison of cases of influenza in cold and hot spots in Yinchuan, 2012−2022

| Group | Towns/streets | Case | $\chi^{2}$ | $P$ |
| --- | --- | --- | --- | --- |
| Hot spots | 16 | 2242 | 656.96 | ＜0.0001 |
| Cold spots | 28 | 980 |  |  |
| Total | 44 | 3222 |  |  |

**Table S5** Clusters identified by the space-time scan statistics on influenza, 2012–2022

| Cluster pattern * | Range of cluster time | Towns/Streets | Coordinates/  radius | Observed  cases | Expected  cases | RR | LLR | $P$-value |
| --- | --- | --- | --- | --- | --- | --- | --- | --- |
| 1 | 2018/1/1 to 2022/12/31 | Changcheng Middle Road | (38.4 N, 106.22E) / 0 km | 967 | 158.44 | 7.72 | 1031.70 | ＜0.0001 |
| 2 | 2019/1/1 to 2019/12/31 | Fenghuang North Street, Jiefang West Street, Wenhua Street, Yuhuangge North Street, Lijing Street, Mancheng North Street, Beijing Middle Road, Shanghai West Road, Fengdeng Town, Xihuayuan Road, Beijing West Road, Zhenbeibu Town, Xigang Town, Nanliangttaizi | (38.55N,106.23E) /11.37 km | 266 | 114.28 | 2.42 | 75.99 | ＜0.0001 |
| 3 | 2014/1/1 to 2014/12/31 | Wutongshu Town | (38.16N,106.26E)/ 0 km | 37 | 2.58 | 14.47 | 64.28 | ＜0.0001 |
| 4 | 2012/1/1 to 2012/12/31 | Chongxing Town | (38.03N,106.35E)/ 0 km | 31 | 5.89 | 5.30 | 26.46 | ＜0.0001 |

1 is the most likely cluster area, and 2-4 is the second possible cluster area.

**Table S6** The results of the spatial regression model -LM test

| Test | Statistic | *P*-value |
| --- | --- | --- |
| SEM |  |  |
| Moran’s *I* | 0.194 | 0.846 |
| LM | 0.048 | 0.827 |
| Robust LM | 7.972 | 0.005 |
| SLM |  |  |
| LM | 1.773 | 0.183 |
| Robust LM | 9.697 | 0.002 |

Note: SEM: spatial error model. SLM: spatial lag model LM: lagrange multiplier

**Table S7** The result of Ordinary Least Squares (OLS) regression analysis

| Variable | Coefficient | t | *P*-value |
| --- | --- | --- | --- |
| vaccine coverage (%) | -1.3558 | -2.77 | ＜0.05 |

**
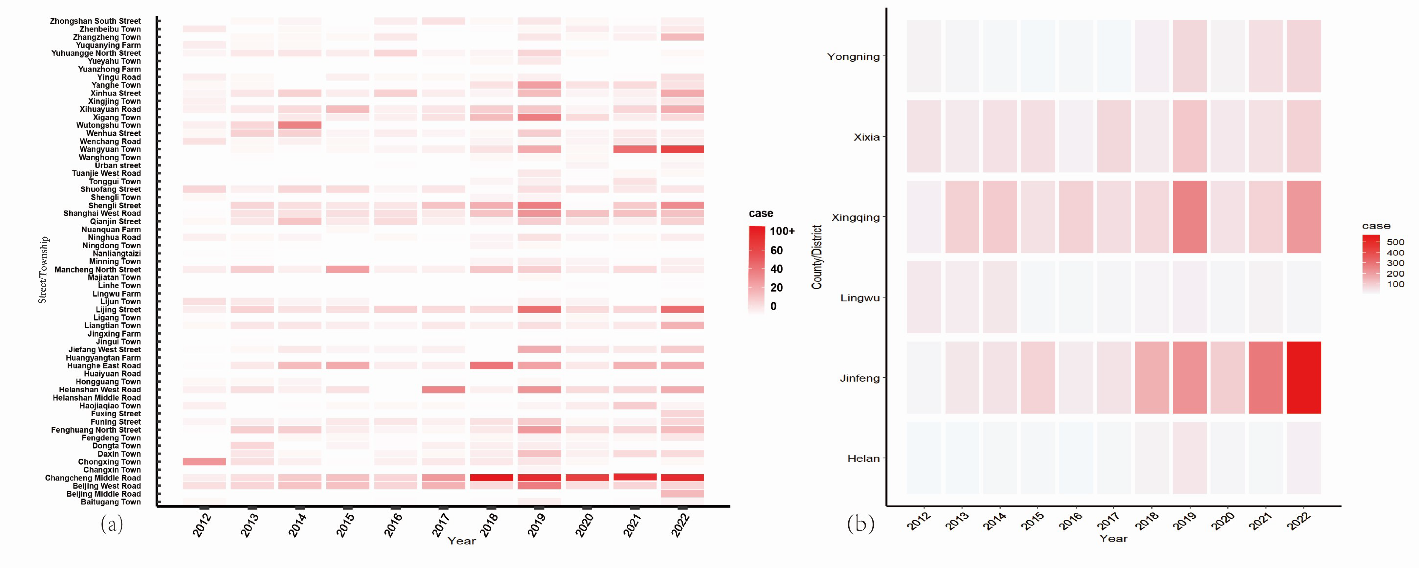
**

**Fig. S1.** Case of influenza in Yinchuan, China, 2012–2022 (/100,000) (a) at the township level (b) at the county level.

**1. Cross-correlation function (CCF) analysis**

Cross-correlation function (CCF) analysis^1^ is commonly employed to study trends, periodicity, or other relationships in two-time series data, providing insights into their mutual influences. In this analysis, a positive value indicates a positive correlation, a negative value indicates a negative correlation, and zero indicates no correlation. Baidu Index serves as a tool based on a vast amount of online user behavior data for measuring online popularity and trends^2^. It collects daily search volumes for specific keywords on the Baidu search engine, providing a visual representation of the public's interest. In our study, we use it to measure the level of public health awareness. Similarly, we use vaccination data from the Yinchuan Center for Diseases Prevention and Control vaccination information system to investigate whether the rebound during the study is related to those factors.

**
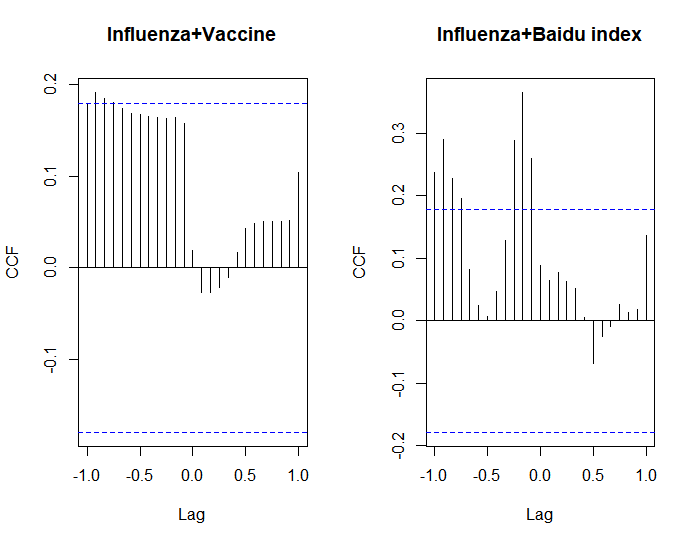
Fig. S2.**  The Cross-correlations between the Influenza cases and Vaccine and the Baidu index from 2012 to 2022


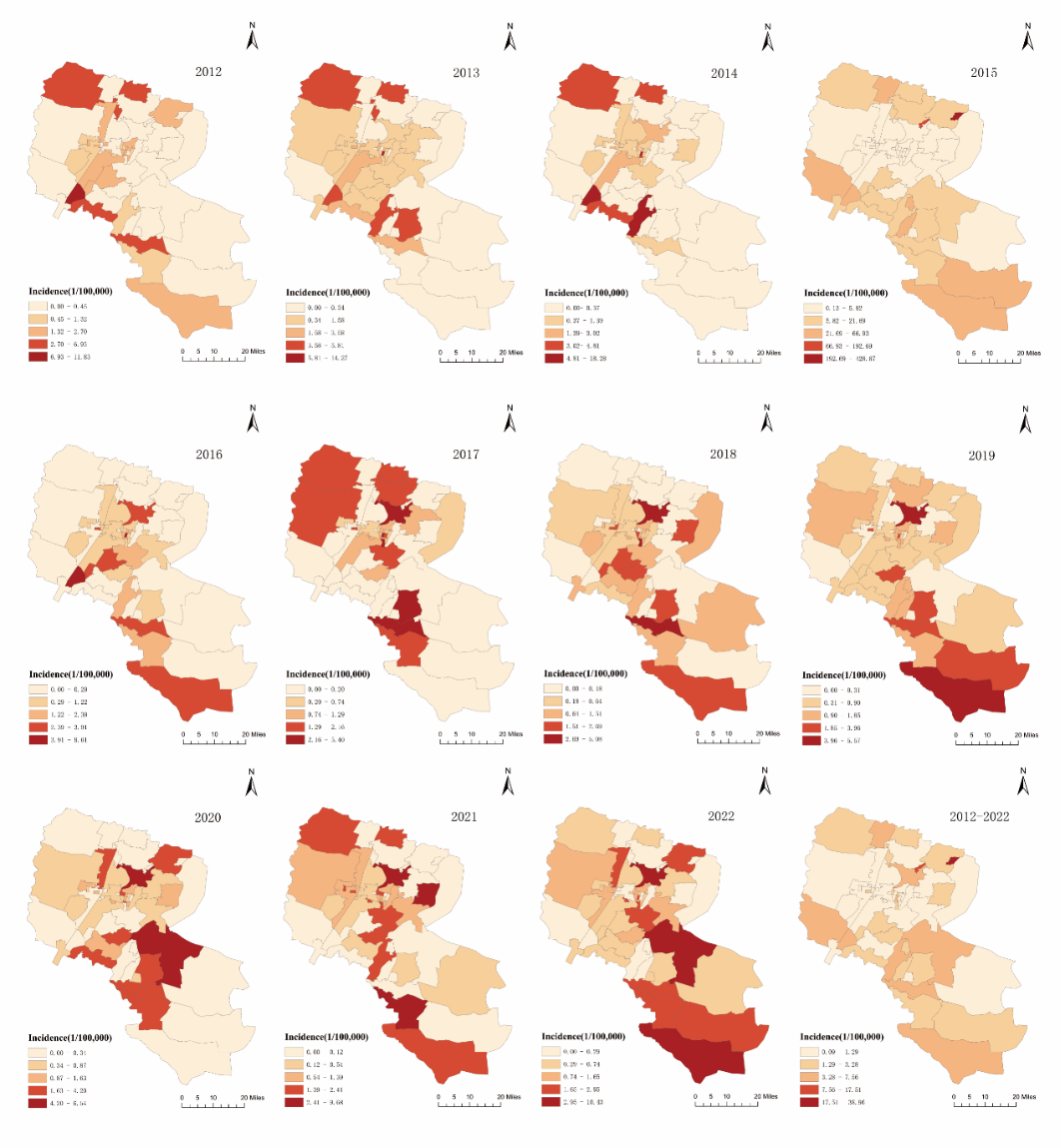
 **Fig. S3.** Annual incidence of influenza at the township level in Yinchuan, China, 2012–2022

In Fig. S3., the annual incidence of influenza focuses more on showcasing the incidence at a specific time point or within a short time window, which might include seasonal fluctuations or short-term changes induced by specific events. Thus, the trends observed on the map might primarily reflect the situation at time point. In contrast, three-dimensional trend surface analysis typically captures trends and patterns over a longer time range. In our study, based on the SMR of influenza from 61 townships in Yinchuan from 2012 to 2022, we calculate the average SMR value for each township as the dependent variable might fit the incidence data over the entire period, revealing long term trends.

**Reference**

1.Cheung, Y. W. & Ng, L. K. A causality-in-variance test and its application to financial market prices. *Journal of Econometrics* **72**, 33-48 (1996).

2.Zhao, Y. *et al.* Analysis of the Transmissibility Change of 2019-Novel Coronavirus Pneumonia and Its Potential Factors in China from 2019 to 2020. *Biomed Res Int* **2020**, 3842470. <https://doi.org/10.1155/2020/3842470> (2020).
